# “Blessed are the Nations with High Levels of Schizophrenia”: National Level Schizophrenia Prevalence and Its Relationship with National Levels of Religiosity

**DOI:** 10.1007/s10943-021-01353-z

**Published:** 2021-08-02

**Authors:** Edward Dutton, Guy Madison

**Affiliations:** 1 Asbiro University, Lodz, Poland; 2 Department of Psychology, Umeå University, Umeå, Sweden

**Keywords:** Religion, Atheism, Schizophrenia, Health, Intelligence

## Abstract

Schizophrenia is correlated with religious delusions but, heretofore, the relationship between schizophrenia prevalence and religiosity has not been explored at the national level. Examining this relationship, we find that national level schizophrenia prevalence is correlated with national level religiosity and strongly negatively correlated with national level atheism across 125 countries. When controlling for cognitive performance and economic development in multiple regression analyses, the proportion of the variance explained was 2.9% (*p* < .005) for Religiousness and 5.1% for Atheism (*p* < .00005). Alternative causal interpretations of this association are discussed.

## Introduction

The extent of belief in God or gods and adherence to religious ideas varies vastly across individuals and between countries. Why is this? Religiosity seems to have a profound influence on peoples’ behaviour and experiences, including on their physical health, psychological well-being, and quality of life, as reviewed by Koenig (2012). Religiosity is, therefore, of great importance and has strong implications for how people conduct themselves in groups and form their societies. Schizophrenia is one factor that has been implicated in this regard, as it is associated with religious delusions and hyper-religiosity.

That such associations exist makes a great deal of theoretical sense. Schizophrenia is conceived of, in part, as *hyper-mentalism.* Mentalising involves being observant of external cues of many kinds, and being interested in deducing people’s mental states from these cues. There is a range of severity to schizophrenia-type conditions. Mild symptoms are summarised as ‘schizoid personality.’ This is characterised by anhedonia and apathy. More severe is ‘schizotypal personality,’ where the schizoid symptoms are accompanied by social anxiety, paranoid ideation, unconventional, or paranoid beliefs and, sometimes, psychosis (Hodgekins, 2015, p. 184). Diagnosable schizophrenia is a particularly severe manifestation of these characteristics (Dowson & Grounds, 2006). Badcock (2003) has argued that schizophrenics practice mentalising to a pathological degree. They are obsessed with such cues and are so hyper-sensitive to them that they read too much into them. This means that a frown could be interpreted as murderous intent, leading to schizophrenics becoming paranoid. This extends to how schizophrenics experience the world. They strongly perceive cues of a mind from everyday observation of the world itself. This heightened propensity to perceive associations and patterns also seems to be associated with creativity, in terms of attaining artistic education (MacCabe et al., 2018) and producing academic publications (Dutton et al., 2020).

Empirically, we find that an individual’s placing on the spectrum that has schizophrenia at one extreme is correlated with experiencing religious delusions, as for example in a study of the Xhosa (Connell et al., 2015), and believing in the paranormal (Thalbourne, 1994) and outlandish conspiracy theories (Barron et al., 2018). See Rogers and Paloutzian (2006) for a review. On the basis that religious delusions are associated with religiosity, we would expect schizophrenia to also be associated with hyper-religiosity. One third of schizophrenics are indeed very strongly involved with their local mainstream church, and a further 10% are involved in small sects that tend to be fervently religious, known as New Religious Movements, according to data from Switzerland (Huguelet et al., 2006). These are much higher proportions than in the general population. A systematic literature review looked at finer grained relationships, such as between schizophrenia and types of religiosity, but, similarly, concluded that hyper-religiosity is a robust correlate of schizophrenia (Grover et al., 2014). It is therefore reasonable to assume that a person’s position on a dimension from the lowest level of schizotypal personality to a schizophrenia diagnosis is to some extent associated with that person’s level of religiousness.

This raises the question of whether this association is also reflected at the group level. There are substantial and robust national differences in how religious people in different countries are (e.g. Zuckerman, 2007). This tendency may also include believing in a metaphysical reality, for example. Furthermore, there are differences in religiosity between ethnic groups within multi-ethnic societies, and these persist even when controlling for factors that might influence religiosity, such as socioeconomic status (Kanazawa et al., 2007; Chatters et al., 2009). Controlling for these factors is important because stress, mortality salience, and feelings of social exclusion have been shown to elevate religiosity (Norenzayan & Shariff, 2008). Schizophrenia also seems to be influenced by stress, at least in individuals with a genetic propensity for this condition (e.g. Gomes & Grace, 2017). It might therefore be argued that hardships such as poverty contributes to country level differences in schizophrenia, which would be consistent with the fact that schizophrenia is less prevalent in developed, wealthy countries (World Health Organisation, 2004, p. 35).

There are nevertheless also pronounced differences in religiosity between socioeconomically and culturally similar countries. For example, based on data from 2007, 68% of Finns claim to believe in God, compared to 45% of Swedes (Dutton, 2014, Ch. 12). Similarly, there are national differences in the prevalence of schizophrenia (e.g. Saha et al., 2005) and, in some cases, there are very substantial differences between neighbouring (and socioeconomically and culturally relatively similar) countries, such as between Sweden and Finland, with the Finnish schizophrenia prevalence being double that of Sweden (Suvisaari et al., 1999). Indeed, Finland provides a natural control for nationality, in that 1.5% of Finns have been diagnosed with schizophrenia compared to 0.7% of Finland’s Swedish-speaking minority (Suvisaari et al., 2014). In the USA, it has been found that African-Americans are twice as likely as Whites to suffer from schizophrenia, even when controlling for socioeconomic factors (Bresnahan et al., 2007). The heritability of schizophrenia is extremely high, at the level of about 0.8 (Ekelund et al., 2000; Hiker et al., 2018). One should therefore expect group differences in schizophrenia to follow ethnic lines, which typically coincide with national states (see Salter, 2007). Thus, assuming that a group’s average level of schizotypy is reflected by its schizophrenia prevalence, we would expect the latter to be associated with its average level of religiousness. This is a reasonable assumption, as it has been shown that the higher people score on the schizotypy scale, the greater is their risk of schizophrenia (Lenzenweger, 2018).

Religiosity has decreased rapidly in the industrialised world in recent centuries, which has largely coincided with its economic development. Across countries, there remains a strong negative correlation between religiosity and level of economic development (for a review, see Lynn et al., 2009). We cannot say to what extent the causality goes in one or the other direction, but because of the rapid secular change it would seem likely that any factors associated with economic development, such as higher level of education, better healthcare, stronger rule of law, and higher levels of security might reduce the need for, and deter people from, religious worship. In any case, we must control for the level of economic development, as mentioned above, with a suitable proxy being per capita gross domestic product (GDP). Furthermore, wealth and income are in turn robustly correlated with intelligence, and intelligence is in turn negatively correlated with schizophrenia (for a meta-analysis, see Mesholam-Gately et al., 2009). This association seems to have common genetic influences (Hagenaars et al., 2016), which suggests it would be very strong at the level of nations, which represent the average across huge numbers of individuals who are typically more genetically similar than across country borders (see Salter, 2007). A negative correlation between religiosity and intelligence might therefore be driven entirely by the association between intelligence and schizophrenia, so we have to control for intelligence.

Thus, we test the hypothesis that there is a positive relationship between religiosity and schizophrenia prevalence at the national level, controlling for economic development and cognitive ability.

## Method

### General Approach and Data Collection Procedures

Data were compiled from several different sources, as described in detail below. There are almost two hundred countries in the world, and an additional few dozen demographic territories that are meaningful units of analysis for the present questions. Several countries and territories do not participate in various measurement programs, however, and data are therefore not available for all countries. Moreover, the number of countries that have complete data decreases the more measures that are required for a particular analysis. Based on the availability of data, we used 125 out of our list of 220 countries and territories. These are found in Table 6 together with the raw variables used in the present analyses. For religiosity, we draw upon two different measures from two different reviews that compile data from the World Values survey, and intelligence is gauged via student achievement test scores. Schizophrenia prevalence estimates come from the World Health Organisation. All of these measures, except the GDP, are based on samples of the country populations that have been selected by the organisations that have measured them, and we refer to these sources for more detailed information about sample sizes and sampling procedures. However, being a cross-sectional design, it cannot inform about causality with regard to the national level religiosity-schizophrenia nexus. Analyses were performed in Statistica v. 7.1 (Statsoft, Inc.) and IBM SPSS Amos v. 21.

### Religiosity and Atheism

Zuckerman (2007) reviewed a number of surveys and other studies reflecting the proportions of people claiming to be religious or otherwise in different countries. He noted a range of potential problems with these data, including low response rates and political climates that may deter respondents from disclosing their atheism or religiosity, and that these factors differ across countries. Nevertheless, data from the World Values Survey, conducted between 1999 and 2002 would seem to be the best available data, according to Lynn et al. (2009). Thus, the data used in the present study were taken from Lynn et al. (2009), who calculated the atheism variable, and from Lynn and Vanhanen (2012), who calculated the religiosity variable, using the following questions in the World Values Survey:

- A006. Religion important. Question: for each of the following aspects, indicate how important it is in your life. [Religion]. Response: very important (%).
- F024. Belonging to a religious denomination. Question: Do you belong to a religious denomination? Response: Yes (%).
- F028. How often do you attend religious services? Question: Apart from weddings, funerals and christenings, about how often do you attend religious services these days? Response: Once a month or more (%).
- F034. Religious person. Question: Independently of whether you go to church or not, would you say you are... Response: A religious person (%).
- F050. Believe in God. Question: Which, if any, of the following do you believe in? [Believe in God]. Response: Yes (%).
- F063. How important is God in your life? Please use this scale to indicate. (10 means very important and 1 means not important at all). Response: codes 7 to 10.

Estimates of the proportion of the population who consider themselves religious were available for 144 countries in total, and for these countries Atheism estimates were missing for Bosnia and Herzegovina, Cyprus, Malta, Myanmar, North Korea, Puerto Rico, and Sudan, which leaves 137 estimates.

### Schizophrenia Prevalence

Schizophrenia prevalence was obtained for 156 countries using the Daily Adjusted Life Year (DALY) measure, which assesses mortality and morbidity, in terms of the number of years of healthy life lost to a particular disease per 100,000 residents. We used a data set from 2004, which seems to be the most recent set of concurrent data for the countries in the present study (World Health Organisation, 2004). The DALY data were preferred because they are generally more comparable than those from the landmark academic meta-analysis of national differences in schizophrenia (Saha et al., 2005), which involved small and incomparable samples. It might be argued that international comparisons of schizophrenia prevalence are difficult to make due to differences in access to healthcare and even national differences in awareness of mental illness. However, developed countries have reasonably more extensive healthcare, which will detect more cases of schizophrenia, so the fact that developed countries report lower rates of schizophrenia than do developing countries speaks against this confound.

### Gross Domestic Product (GDP) Per Capita

GDP was extracted from the United Nations statistics website, and we downloaded the most recent figures from 2018, in US dollars, for 212 countries (United Nations, 2020).

### Mean National Level Intelligence Estimates

We used the World Bank’s National Harmonised Test Scores (NHTS) as a proxy for national levels of intelligence (Patrinos & Angrist, 2018). The best source of national IQ estimates is the work of Lynn and Becker (2019), which compiles and critically evaluates a large body of empirical studies, building on previous reviews (Lynn & Vanhanen, 2002, 2012). However, these estimates do not exist for all countries, which can limit their utility when global relationships are considered. Lynn and Becker report estimates for 168 countries in total, 113 of which overlap with countries that we also have religiousness data for. NHTS data were available for 175 countries, 120 of which overlap with the religiousness data. The validity of the NHTS as a proxy for intelligence is supported by a Pearson correlation of 0.854 with the Lynn and Becker data for the 109 countries that had estimates for both.

### Control for Spatial Autocorrelation

There are likely many factors that may influence the key variables schizophrenia and religiousness and atheism other than the ones included in our analyses. Many of these are associated with a country’s geographical location, through climate, culture, and other environmental influences. We control, to some extent, for such spatial autocorrelation by categorising the countries into regions identified to account for such major differences by anthropological research, Specifically, each country was dummy-coded into one of Murdock’s (1949) six regions, namely Africa, Insular Pacific, East Eurasia, North America, South America, and West Eurasia.

## Results

The availability of data differed across the countries: GDP (212), Schizophrenia (156), NHTS (175), Religiousness (144), and Atheism (137). Because the pattern of available data varied across variables, only 116 countries had complete data. A meaningful analysis should include at least Schizophrenia and one index of religiosity. This condition was fulfilled for 125 countries for Religiousness and 121 countries for Atheism, as no country with an Atheism estimate lacked a Religiousness estimate. The selected countries and their raw values for each of the five variables are listed in Table 6 in the Appendix.

Table 1 lists the descriptive statistics across countries, showing that for all variables except GDP and Atheism, the raw values were sufficiently normally distributed to fulfil the assumptions behind the parametric analyses, with skewness and kurtosis estimates within ± 2.0 (George & Mallery, 2010). This could be achieved also for GDP and Atheism, by taking the natural logarithm of GDP and the inverse of Atheism ((1 + X)^−0.5^). The variables and their transformations used in the following analysis are marked in bold in Table 1.

Table 2 shows that all Pearson zero-order correlations between the selected variables exhibit medium to strong correlations, which were all statistically significant.

**Table 1:** Descriptive statistics, including transformations

	*N*	*M*	Min	Max	SD	Skewness	Kurtosis
GDP per capita ($)	125	15,354	99.57	82,708.51	20,341.4	1.701	2.112
Log GDP per capita	**125**	**8.65**	**4.60**	**11.32**	**1.55**	**− 0.0925**	**− 0.894**
NHTS	**121**	**428.13**	**304.97**	**575.3**	**64.05**	**0.132**	**− 0.994**
Religiousness (%)	**125**	**86.74**	**44.60**	**99.50**	**14.14**	**− 1.106**	**0.3428**
Atheism (%)	121	9.24	0.50	64.00	14.05	1.980	3.473
Inverse Atheism	**121**	**0.54**	**0.124**	**0.820**	**0.260**	**− 0.232**	**− 1.627**
Schizophrenia	**125**	**239.96**	**164.25**	**321.87**	**36.06**	**− 0.0865**	**− 0.514**

Variables used in the analyses are bold

**Table 2:** Pearson zero-order correlation matrix for all selected variables

		2	3	4	5
1	Religiousness	0.872	0.592	**− **0.749	**− **0.616
2	Inverse Atheism	–	0.639	**− **0.790	**− **0.686
3	Schizophrenia		–	**− **0.576	**− **0.476
4	NHTS			–	0.803
5	Log GDP per capita				–

All *p* < .00001. All *N* = 125 except those involving Atheism *N* = 121

To account for the unique association between Schizophrenia and the two religiosity measures, one multiple regression was computed for each of these, as seen in Table 3. When combined together, the four predictor variables apparently account for 56.7% and 67.4% of the variance, for Religiousness and Inverse Atheism, respectively. While the contribution of GDP is negligible and also non-significant, Schizophrenia seems to make a small but significant contribution, even though it is dwarfed by the large effect of NHTS. We computed the squared semi-partial correlations as estimates of the unique variance, indicating unique contributions of between 3 and 5%.

**Table 3:** Results of linear regressions of Schizophrenia, NHTS, GDP, and Murdock region upon the two religiosity measures

	*β*	*r*^2^	*p*
*Regression summary for Religiousness*^a^			
Intercept			< .00001
Schizophrenia	0.207	0.029	< .005
NHTS	**− **0.553	0.105	< .00001
Log GDP per capita	**− **0.098	0.0034	= .33
Murdock region	0.017	0.0005	= .81
*Regression summary for Inverse Atheism*^b^			
Intercept			< .00001
Schizophrenia	0.276	0.051	< .00005
NHTS	**− **0.516	0.080	< .00001
Log GDP per capita	**− **0.098	0.003	= .30
Murdock region	**− **0.078	0.004	= .22

Note. *r*^2^ = squared semi-partial correlations, ^a^*N* = 125, *R* = .740, *R*^2^ = .548, Adjusted *R*^2^ = .537 ^b^*N* = 121, *R* = .828, *R*^2^ = .685, Adjusted *R*^2^ = .674

Apparently, NHTS also assumes most of the variance associated with GDP, rendering its actual associations with Religiousness and Atheism non-significant. The Variance Inflation Factors ranged from 0.419 to 2.90 for Religiousness and from 0.314 to 3.28 for Inverse Atheism. As that is less than 5, we conclude that there is no substantial multicollinearity (Kutner et al., 2005). Furthermore, we estimated the skewness of the residual model co-variances to fall between those two values in all cases in order to satisfy the basic requirement for regression analysis.

We mentioned in the Introduction that associations have been found between schizophrenia and stress (e.g. Gomes & Grace, 2017), which might arguably be induced by poverty and its associated conditions. As the regression models run so far do not speak directly to this issue, we ran a third regression to specifically test the association between Schizophrenia and GDP, controlling for all the other variables. This model explained around 35% of the variance, as seen in Table 4, which is substantially less than the 55–67% for the models that predict Religiousness through all the other variables. This model, moreover, exhibited significant associations only between schizophrenia and inverse atheism, the latter of which apparently assumed most of the variance associated with religiousness.

**Table 4:** Regression summary for Schizophrenia

	*β*	*r*^2^	*p*
Intercept			< .00001
Religiousness	**− **0.184	0.008	= .22
Inverse Atheism	0.554	0.060	< .001
NHTS	**− **0.178	0.007	= .24
Log GDP per capita	**− **0.130	0.005	= .31
Murdock region	**− **0.092	0.006	= .28

Note. *N* = 124, *R* = .616, *R*^2^ = .379, Adjusted *R*^2^ = .353, *r*^2^ = squared semi-partial correlations

**Fig. 1:**
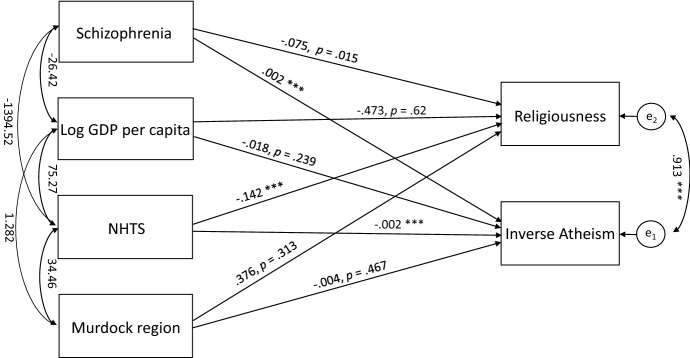
Path model. Note. All estimates are unstandardized

To further elucidate these rather complex associations, we conducted a path analysis to incorporate both measures of religiosity in the same model and assess the overall model fit. Several different models were estimated, and that which produced the best model fit is depicted in Fig. 1, namely *χ*^2^(1) = 1.33, *ns*, CFI = 0.99, RMSEA = 0.051, 90% CI = 0.000, 0.249. Again, both Schizophrenia and NHTS exhibit highly significant paths to both Atheism and Religiousness, even when the two latter occur in the same model. Atheism and religiousness are associated close to unity, while the associations with Atheism appear again to be stronger than those to Religiousness, as seen in Table 5, listing the model parameters.

**Table 5:** Path analysis coefficients

			Estimate	*SE*	*CR*	*p*
Inverse Atheism	←	Murdock region	**− **.004	.006	**− **.728	.467
Inverse Atheism	←	Schizophrenia	.002	.000	4.099	***
Inverse Atheism	←	Log GDP per capita	**− **.018	.016	**− **1.176	.239
Inverse Atheism	←	NHTS	**− **.002	.000	**− **5.212	***
Religiousness	←	Log GDP per capita	**− **.473	.956	**− **.495	.620
Religiousness	←	NHTS	**− **.142	.025	**− **5.754	***
Religiousness	←	Murdock region	.376	.373	1.008	.313
Religiousness	←	Schizophrenia	.075	.031	2.432	.015
Schizophrenia	↔	Log GDP per capita	**− **26.418	5.095	**− **5.186	***
Log GDP per capita	↔	Murdock region	1.282	.310	4.132	***
Log GDP per capita	↔	NHTS	75.272	10.512	7.161	***
Schizophrenia	↔	NHTS	**− **1394.527	232.734	**− **5.992	***
Murdock region	↔	NHTS	34.464	11.285	3.054	.002
e_1_	↔	e_2_	.913	.151	6.036	***

Note. *CR* critical ratio, *SE* standard error, ***p < .00001, path coefficients are unstandardized estimates

Overall, the path model renders additional support to the direct effect of Schizophrenia on religiosity. Models with paths from GDP to Schizophrenia exhibited substantially worse fits, which together with the relative strengths of the associations speaks against any significant causal link between these two variables.

## Discussion

Our key finding is that schizophrenia prevalence is substantially correlated with two different measures of religiosity, namely Religiousness (positively) and Atheism (negatively), and that meaningful associations remain even after controlling for GDP, NHTS, and spatial autocorrelation. The fact that Religiousness is correlated with Inverse Atheism at 0.87 likely reflects the distinction between not believing in God and actively believing that there is no God, a belief known as ‘hard atheism.’ We further conclude that this association is not substantially driven by an association between schizophrenia and GDP, with GDP being a proxy for poverty or stress. The third regression model, which tested the association between schizophrenia as the dependent variable, and all other variables as predictors, explained substantially less variance than the models that pitted the religiosity indicators against all other variables. This indicates that wealth, or its inverse poverty, is not mainly driving the associations between religiosity and schizophrenia, for example through some confound with country level differences in development or cultural factors. This has also been shown when making inter-racial comparisons and controlling for key environmental variables such as socioeconomic status (Bresnahan et al., 2007).

## Limitations

As noted above, religiosity becomes elevated at times of stress, and it has also been proposed that this is the case also for symptoms of schizophrenia (Corcoran et al., 2002). Indeed, a number of analyses have demonstrated that periods of stress seem to induce schizophrenia symptoms in those with a genetic propensity towards the condition (e.g. Gomes & Grace, 2017). On the one hand, this would be consistent with our finding that schizophrenia prevalence correlates with national GDP at -0.47 and the fact that schizophrenia is far less prevalent in developed, wealthy countries (World Health Organisation, 2004). On the other hand this interpretation not supported by the models in Tables 4 and 5, nor by the very high heritability of schizophrenia. For example, the heritability of schizophrenia was estimated to 0.79 in Denmark (Hiker et al., 2018) and 0.83 in Finland (Ekelund et al., 2000). This implies that national differences in schizophrenia are unlikely to be substantially explained by stress, due to poverty. Rather, many researchers argue that schizophrenia, being strongly genetic, leads to poverty (e.g. Jeffries et al., 1990, p. 4).

It is possible that although the disposition for schizophrenia is strongly genetic, the condition is only induced in certain conditions, such as those of poverty or high stress. This is consistent with the finding that the effects of environmental factors on schizophrenia decrease to very small levels when genetics is controlled for (Moffitt & Caspi, 2006, p. 67). For a review of gene-environment interaction effects in schizophrenia, see Moran et al. (2016).

Similarly, there may also be a direct environmental link such that the suffering that schizophrenia inflicts may make those individuals more likely to turn to religion for comfort. However, the average proportion of national populations that are religious is in the order of 85 to 90%, while the average point prevalence of schizophrenia is about 0.28% (Charlson et al., 2018). It is extremely unlikely that this small proportion of individuals would be able to have an impact on the religiousness estimates, even if every one of them actually were religious. Rather, as discussed above, we argue that schizotypy is the driving factor, which is in turn reflected in schizophrenia.

Further, making assertions at the population or country level about the relationship between schizophrenia and religiosity also risks the ‘ecological fallacy,’ whereby a relationship that holds at the individual level is not necessarily true at the group level. For example, at the individual level religiousness is robustly correlated with physical health (Koenig, 2012). However, countries that have relatively *poor* public health tend to be more religious (Lynn & Vanhanen, 2012). That said, it is a matter of debate whether religiousness makes you healthier or whether religiousness and sound health are expressions of some underlying factor (see Dutton et al., 2018). If, however, schizophrenia causes people to be more religious at the individual level, then we would expect that a group of individuals with a higher schizophrenia prevalence would, on average, be more religious than a group of individuals with a lower schizophrenia prevalence.

In conclusion, we must ask ourselves which interpretation with regard to the national level religiosity-schizophrenia nexus is the most parsimonious. Is schizophrenia a partial cause of national differences in religiosity or not a cause, meaning that the nexus is entirely mediated by some other factor? To this, we submit that differences in schizophrenia prevalence are likely to be substantially genetic in origin, and that the available evidence suggests the simplest explanation is that schizophrenia is a causal factor in national differences in religiosity. That said, we reach this conclusion with caution, due to the nature of the data at hand.

## Appendix

See Table 6.

**Table 6:** List of all countries included in the analyses, with their raw data

Country	Murdock region	GDP	Religiousness	Atheism	Schizophrenia	NHTS
Afghanistan	EastEurasia	551.865	99.5	0.5	253.778	354.7588
Albania	WestEurasia	5223.809	75.8	8.0	247.412	434.1276
Algeria	Africa	4114.706	91.9	0.5	239.752	374.0891
Angola	Africa	3437.295	98.0	1.5	252.602	325.9655
Argentina	SouthAmerica	11,687.6	88.2	4.0	253.404	408.1726
Armenia	WestEurasia	4212.133	81.6	14.0	269.694	442.9695
Australia	InsularPacific	58,392.71	70.5	25.0	164.255	515.6854
Austria	WestEurasia	51,230.28	77.4	18.0	185.116	507.6394
Azerbaijan	EastEurasia	4717.72	85.3	0.5	269.573	415.9462
Bangladesh	EastEurasia	1670.796	96.7	0.5	265.704	368.3153
Belarus	WestEurasia	6311.718	57.7	17.0	206.098	488.1431
Belgium	WestEurasia	47,292.97	62.3	43.0	186.130	516.8079
Benin	Africa	906.5387	99.3	0.5	244.670	383.9229
Bolivia	SouthAmerica	3548.59	98.4	1.0	253.353	
Bosnia and Herzegovina	WestEurasia	5951.383	93.0		241.524	416.1341
Botswana	Africa	8258.227	99.4	0.5	234.546	391.3183
Brazil	SouthAmerica	8920.7	93.5	1.0	255.328	413.2448
Brunei Darussalam	EastEurasia	31,627.23	90.1	0.5	312.101	437.5159
Bulgaria	WestEurasia	9387.813	66.6	34.0	238.471	441.0933
Burkina Faso	Africa	820.1744	99.1	0.5	246.534	403.6543
Burundi	Africa	293.9633	99.4	0.5	242.958	422.7478
Cambodia	EastEurasia	1512.127	74.2	7.0	274.896	451.8896
Cameroon	Africa	1534.492	99.3	0.5	244.614	378.8688
Canada	NorthAmerica	46,192.38	74.5	22.0	185.942	533.998
Chad	Africa	735.7297	99.1	0.5	246.930	333.1113
Chile	SouthAmerica	15,923.36	85.0	2.0	254.046	452.2182
Colombia	SouthAmerica	6649.636	92.9	1.0	253.524	419.0275
Congo-Brazzaville	Africa	2702.559	97.4	2.7	241.964	370.6141
Costa Rica	NorthAmerica	20,705.21	98.4	1.0	252.764	428.5557
Croatia	WestEurasia	14,674.02	86.6	7.0	187.935	487.5812
Cyprus	WestEurasia	28,967.58	95.6		273.036	502.1622
Czechia	WestEurasia	22,992.06	43.6	61.0	185.826	512.2216
Denmark	WestEurasia	61,833.71	62.0	48.0	187.542	517.8778
Dominican Republic	NorthAmerica	7650.09	87.0	7.0	254.906	345.2165
Ecuador	SouthAmerica	6344.872	98.5	1.0	253.573	420.1486
Egypt	Africa	2537.512	95.4	0.5	273.441	355.9865
El Salvador	NorthAmerica	4058.243	93.8	1.0	254.538	435.9228
Estonia	WestEurasia	23,241.89	47.1	49.0	201.730	543.2061
Ethiopia	Africa	735.1145	99.4	0.5	238.310	348.199
Finland	WestEurasia	50,135.72	68.4	28.0	187.690	533.7076
France	WestEurasia	41,358.09	56.4	44.0	189.100	510.2606
Gambia	Africa	716.1203	99.2	0.5	244.150	352.8996
Georgia	EastEurasia	4396.667	82.9	4.0	235.620	399.7663
Germany	WestEurasia	47,513.7	58.4	42.0	185.760	517.2814
Greece	WestEurasia	20,731.2	83.0	16.0	185.063	468.6362
Guinea	Africa	937.5969	99.3	0.5	247.000	408.2491
Honduras	NorthAmerica	2500.11	98.7	1.0	256.040	399.7508
Hungary	WestEurasia	16,264.02	64.7	32.0	206.746	495.4812
Iceland	WestEurasia	76,867.3	78.2	16.0	184.830	497.6828
India	EastEurasia	2054.757	90.4	3.0	268.900	399
Indonesia	EastEurasia	3893.493	91.9	1.5	321.870	394.9151
Iran	EastEurasia	5783.495	94.3	4.5	275.670	432.1082
Iraq	EastEurasia	5523.079	99.3	0.5	279.360	363.4325
Ireland	WestEurasia	79,414.6	88.0	5.0	185.620	521.3286
Israel	EastEurasia	44,214.91	84.2	15.0	188.000	480.7522
Italy	WestEurasia	34,388.51	79.5	6.0	185.580	492.9878
Jamaica	NorthAmerica	5354.253	96.4	3.0	254.050	387.133
Jordan	EastEurasia	4237.796	94.4	0.5	273.290	429.9541
Kazakhstan	EastEurasia	9789.504	69.9	12.0	210.640	416.1546
Kenya	Africa	1710.475	99.4	0.5	234.970	454.9584
Kuwait	EastEurasia	34,248.84	98.7	0.5	269.360	383.4022
Kyrgyzstan	EastEurasia	1283.756	77.3	7.0	279.940	420.0865
Lao	EastEurasia	2542.49	84.0	5.0	287.170	368.1421
Latvia	WestEurasia	17,851.58	63.6	20.0	203.750	503.8698
Lebanon	EastEurasia	8223.658	96.2	3.0	275.760	389.889
Liberia	Africa	440.2523	98.7	0.5	247.210	331.7459
Libya	Africa	5147.334	99.3	0.5	273.500	
Lithuania	WestEurasia	19,082.52	78.1	13.0	205.790	495.8863
Madagascar	Africa	527.3966	99.3	0.5	244.010	350.7733
Malawi	Africa	396.6315	99.3	0.5	237.360	359.4841
Malaysia	EastEurasia	11,373.35	91.3	0.5	314.190	445.6753
Mali	Africa	900.0966	99.4	0.5	248.110	307.365
Malta	WestEurasia	33,122.8	92.8		184.870	474.435
Mauritania	Africa	1730.439	99.4	0.5	242.680	342.0936
Mexico	NorthAmerica	9694.852	91.5	4.5	245.990	430.0651
Mongolia	EastEurasia	4103.687	72.9	20.0	270.020	434.6187
Morocco	Africa	3272.948	95.8	0.5	273.580	380.4052
Mozambique	Africa	498.9407	97.2	5.0	239.190	368.2436
Nepal	EastEurasia	990.5566	99.3	0.5	265.390	368.5576
Netherlands	WestEurasia	53,583.14	61.5	42.0	186.280	519.6346
New Zealand	InsularPacific	43,836.15	71.4	22.0	193.700	519.7484
Niger	Africa	571.2738	99.4	0.5	247.160	304.9222
Nigeria	Africa	2153.526	98.5	0.5	246.670	309.025
Norway	WestEurasia	81,335.65	64.1	31.0	187.490	513.5878
Oman	EastEurasia	19,072.78	99.1	0.5	270.000	423.5134
Pakistan	EastEurasia	1330.386	96.0	0.5	266.340	338.6566
Paraguay	SouthAmerica	5794.576	98.6	1.0	252.610	385.5443
Peru	SouthAmerica	6947.25	93.4	1.0	253.060	415.0254
Philippines	EastEurasia	3102.727	88.6	0.5	317.070	361.646
Poland	WestEurasia	15,444	91.7	3.0	233.870	530.0858
Portugal	WestEurasia	23,477.73	85.9	4.0	186.250	508.5149
Republic of Moldova	WestEurasia	2791.034	71.8	6.0	202.680	438.6209
Romania	WestEurasia	12,280.84	87.4	4.0	237.220	442.1617
Russian Federation	WestEurasia	11,394.14	58.1	27.0	206.910	497.5462
Rwanda	Africa	773.0471	99.1	0.5	240.850	358.084
Saudi Arabia	EastEurasia	23,217.2	98.9	0.5	270.200	398.9689
Senegal	Africa	1501.721	99.3	0.5	244.410	412.4539
Sierra Leone	Africa	536.1105	99.3	0.5	250.550	315.8759
Singapore	EastEurasia	62,720.88	74.0	13.0	311.870	575.2722
Slovakia	WestEurasia	19,430.79	76.5	17.0	233.540	485.35
Slovenia	WestEurasia	26,005.1	67.6	35.0	187.200	520.809
Somalia	Africa	99.57643	99.4	0.5	234.840	
South Africa	Africa	6369.232	91.1	1.0	240.510	342.764
Spain	WestEurasia	30,405.83	76.8	15.0	186.360	506.6212
Sri Lanka	EastEurasia	4189.688	98.3	0.5	312.270	400
Sudan	Africa	1208.441	98.9		234.490	379.6337
Sweden	WestEurasia	55,766.81	45.3	64.0	186.010	519.3618
Switzerland	WestEurasia	82,708.51	74.9	17.0	188.710	515.139
Syria	EastEurasia	981.3018	97.8	0.5	277.300	
Tajikistan	EastEurasia	826.6202	89.3	2.0	266.300	390.5656
Thailand	EastEurasia	7273.565	88.0	0.5	315.530	426.6
Togo	Africa	654.7589	99.4	0.5	244.320	383.7178
Trinidad and Tobago	SouthAmerica	17,130.34	94.4	9.0	254.960	458.2889
Tunisia	Africa	3449.581	99.3	0.5	273.840	384.0787
Turkmenistan	EastEurasia	6964.613	89.8	2.0	264.630	
Uganda	Africa	704.3946	96.5	0.5	236.840	397.1597
Ukraine	WestEurasia	2956.922	67.1	20.0	209.780	478.1733
United Kingdom	WestEurasia	42,526.44	64.7	41.5	185.180	520.3564
United Republic of Tanzania: Mainland	Africa	1044.062	97.3	0.5	239.340	388.4802
United States	NorthAmerica	62,917.94	86.8	10.5	185.620	511.7987
Uruguay	SouthAmerica	17,278.06	68.2	12.0	257.200	437.6966
Uzbekistan	EastEurasia	1554.98	81.7	4.0	286.940	474.0751
Yemen	EastEurasia	935.8881	99.2	0.5	275.110	321.327
Zambia	Africa	1572.344	99.3	0.5	238.150	358.1404
Zimbabwe	Africa	1683.766	96.1	4.0	238.167	396.1388
